# Clinical practice guideline recommendations can promote or undermine health equity

**DOI:** 10.1002/gin2.70011

**Published:** 2025-03-28

**Authors:** Nav Persaud

**Affiliations:** ^1^ University of Toronto Department of Family and Community Medicine Toronto Ontario USA; ^2^ St Michael's Hospital in Unity Health Toronto Toronto Ontario USA; ^3^ MAP Centre for Urban Health Solutions St Michael's Hospital in Unity Health Toronto Toronto Ontario USA

**Keywords:** clinical practice guideline, guidelines methodological topics and challenges, guidelines methodological topics and challenges, diversity, equity, and inclusion in guidelines

## Abstract

While clinical practice guideline makers and methodologists signal the importance of considering inequities, recommendations often have little or nothing to do with fairness. Since inequities are, by definition avoidable, guidance on clinical practice is a prime opportunity to make health care more fair. Equity should be a central consideration when deciding who to involve in the guideline process, the guideline's scope, the type of information to consider, how to make recommendations and how to share recommendations. Guideline producers should select topics where guidance can actually address inequities and then use information about disparities to make helpful recommendations. Funders and journal editors should insist that guidelines explain how panels were formed and why new guidance on the topic is needed. Changes to clinical practice guidelines will not be enough to promote health equity, but guidelines can be part of the solution if they are thoughtfully produced and acted on by clinicians and by governments.

## Key messages

- While clinical practice guideline makers and methodologists signal the importance of considering inequities, recommendations often have little or nothing to do with fairness.
- Guideline producers should select topics where guidance can actually address inequities and then use information about disparities to make helpful recommendations.
- Funders and journal editors should insist that guidelines explain how panels were formed and why new guidance on the topic is needed as, in some cases, equity might be advanced by cancelling planned guidelines.

## 1 CLINICAL PRACTICE GUIDELINE RECOMMENDATIONS CAN PROMOTE OR UNDERMINE HEALTH EQUITY OR FAIRNESS

While clinical practice guideline makers and methodologists signal the importance of considering inequities (or avoidable disparities in health outcomes),^1^ recommendations often have little or nothing to do with fairness.^2^, ^3^ The introduction and methods sections of guideline documents routinely mention equity – within a list of other considerations – while recommendations do not relate to equity (see examples in Box 1). Since inequities are, by definition avoidable, guidance on clinical practice is a prime opportunity to make health care more fair.

Box 1. **Examples of passing mentions of equity in clinical practice guidelines where recommendations do not clearly address equity**

Equity mentioned in methods but not in recommendations

A living WHO guideline on drugs for covid‐19: “While the GDG takes an individual patient perspective in making recommendations, it also considers resource implications, acceptability, feasibility, equity, and human rights.”

https://www.bmj.com/content/370/bmj.m3379.long

American Society of Hematology 2020 guidelines for management of venous thromboembolism: treatment of deep vein thrombosis and pulmonary embolism: “…Reductions in health equity for ≥1 of these groups of patients may be present for all of the recommendations considered in this guideline document.” https://www.ncbi.nlm.nih.gov/pmc/articles/PMC7556153/

An Official ATS/ERS/JRS/ALAT Clinical Practice Guideline: Treatment of Idiopathic Pulmonary Fibrosis: “The shortage of organs is a universal problem, and the decision to give bilateral lung transplantation to a single patient rather than give single‐lung transplantation to two patients, including the effect on health inequity, must be considered.”

https://pubmed.ncbi.nlm.nih.gov/26177183/

Treatment of Hypercalcemia of Malignancy in Adults: An Endocrine Society Clinical Practice Guideline: “It was anticipated that resources, cost‐effectiveness, and equity would likely vary, but the treatment would probably be feasible and accessible”

https://pubmed.ncbi.nlm.nih.gov/36545746/

When the effects of recommendations are difficult to predict, a complexity perspective can be used to decide who to involve in the guideline process, the guideline's scope, the type of information to consider, how to make recommendations and how to share recommendations.^4^ In this article, these aspects of guideline development are applied to equity and then tied together using the cliff analogy that shows how to prioritize interventions.^5^ Specific examples are mentioned to illustrate how equity can be sidelined or appropriately centered.

## 2 WHO TO INVOLVE IN THE GUIDELINE

Involving patients and members of the public can have important effects on the process and resulting guidance,^6^ so it is important to ensure that those involved reflect the diversity of the populations affected by the guidance.^7^ Indeed, involving members of the public unfairly could actually exacerbate inequities compared with not involving them at all. Guideline producers rarely describe their processes for recruiting panel members who are involved in developing recommendations and panels are dominated by white men.^3^ Fair processes to comprise guideline panels could represent one step toward orienting guideline content toward equity. At the least, more diverse guideline panels could avoid signaling that some are valued more than others within health care.

## 3 SCOPE AND FOCUS

Topics selected for clinical practice guidelines often recapitulate rather than address health inequities. Increasing prescribing of opioids for chronic non‐cancer pain has fueled a crisis of deaths but while there are multiple guidelines that recommend opioids for chronic non‐cancer pain, there are few on how to prevent opioid deaths. Opioid exposure and subsequent dependence can occur during emergency department visits, yet guidelines sometimes exclude these types of encounters.^8^ Additionally, the risk of an opioid related death is substantially elevated just after release from incarceration and yet there is little guidance on how to manage this relatively common event.^9^ Opioid‐related deaths are one example of how guideline production can track priorities of pharmaceutical companies that neglect certain conditions even if the companies do not directly fund guidelines. In contrast, there are dozens of guidelines on vitamin D supplementation, an intervention that has repeatedly failed to show any benefit across a broad range of uses and outcomes.^10^ Recent guidelines recommend a variety of new inhalers for COPD and exquisitely expensive cancer treatments but how many represent a substantial advance over older treatments?^11^, ^12^

Proven interventions that are well suited to address inequities exist and yet recommendations that could promote equity are relatively rare. For example, HPV self‐testing obviates the need to book an appointment for a pelvic examination by a healthcare provider that is required with cytological Pap smears. HPV self‐testing can help improve cervical cancer screening rates and detection rates of high risk lesions^13^ and yet some guidelines do not mention HPV self‐testing.^14^

Special outreach might be appropriate for recommended interventions such as phone calls for individuals due for cancer screening,^15^ and guideline documents could provide advice on how to equitably implement recommendations. Although discriminatory practices within health care have been described,^16^ few clinical practice guidelines address how to deliver care fairly and without discrimination.

Social interventions can be implemented in clinical settings. There is evidence that connecting children experiencing poverty and women experiencing intimate partner violence with appropriate supports through clinical encounters improves health and avoids harm.^17^ But such social interventions are often not treated as clinical topics although the interventions are implemented by clinicians in clinical settings.

Clinical practice guidelines can help to advance access to care that might be deemed controversial or even illegal in some settings such as gender‐affirming care and reproductive care. Recommending proven health care interventions could both directly help individuals facing discrimination and help to foster wider societal changes.

## 4 TYPES OF INFORMATION USED IN GUIDELINES

Guideline producers understandably tend to focus resources on identifying evidence of the effectiveness of interventions since this information is needed to make recommendations. Recommendations sometimes fail to incorporate Information about inequities, even in clinical areas with obvious inequities such as Hepatitis C that disproportionately affects people who use drugs and men who have sex with men.^18^ Guidance on how to ensure people at risk for Hepatitis C are tested and treated might help to address inequities. Evidence about the effects of interventions should be combined with evidence of disparities in health outcomes and in the process of care.^19^ For example, disparities in cardiovascular disease outcomes could be a reason in favor of interventions for the general population (on the assumption that they would disproportionately benefit those experiencing disadvantages) or for specific disadvantaged groups (see the examples of polypills below).

Guideline producers should not expect every intervention to be studied in every population. As is virtually always the case, guideline developers will have to consider the extent to which effects observed in study populations apply to actual patient populations based on factors such as the mechanism of action.^20^ Health inequities are related to social factors and not biological differences such as presumed biological differences based on the social concept of race.^21^ It will often be reasonable to assume that effective interventions, such as treatments for hypertension, will be effective in disadvantaged populations and thus disproportionately benefit them. Indeed, the assumption that interventions work across the population implicitly lies beneath most current recommendations that apply to the general population.

Just as it is important to consider the cost and resource implications of recommendations, it is also vital to keep in mind the actual implications of negative recommendations. The “time needed to screen” to benefit one patient has been cited as a reason to recommend against screening for depression.^22^ But rough estimates of the time implications of clinical practice guidelines often lead to fanciful statistics that do not represent what actually happens in practice.^22^ Some might argue that negative recommendations for interventions of questionable value will “free up” clinician time for other pursuits such as promoting health equity, but there is no specific reason to believe that negative recommendations lead to improvements in care for anyone. Information that could lead to negative recommendations for interventions that promote equity should be scrutinized like other information and back of the envelope estimates of resource implications could be disregarded altogether.

## 5 GUIDELINE RECOMMENDATIONS

Recommendations against routinely providing an intervention can exacerbate inequities. For example, recommendations *against* depression screening assume that routine care will involve clinical evaluations that identify patients who need treatment.^23^ But disparities in access to care, including primary care, mean that some patients will be less likely to be assessed.^24^ Biases in the way care is provided could also lead to disparities in the rate of diagnosis.^25^ Indeed, routine screening for depression in primary care improves the rate of detecting depression overall and reduces disparities.^26^

Screening and other interventions applied across the population could address inequities by disproportionately benefitting those who are less likely to receive appropriate care. Polypills prevent cardiovascular disease and reduce all‐cause mortality but there is not clear guidance on who should receive them and guidelines on the primary prevention of cardiovascular disease typically do not mention polypills.^27^

## 6 SHARING OF RECOMMENDATIONS

Clinicians, the usual intended audience of recommendations, can sometimes play an important role in promoting health equity but often broader changes need to be implemented by governments. Clinical practice guidelines should, where relevant, include systemic changes needed to promote health equity. For example, building more computed tomography scanners outside of cities to implement lung cancer screening.^28^

Clinical practice guidelines that do not actually consider or address inequities in their guidance should not state that they do. Vague mentions of equity do not address inequities, and misleading mentions of equity could give the false impression that inequities are being addressed. Guidance on creating recommendations should be revised to avoid a “checkbox” approach where guideline producers feel pressure to mention equity, among a list of other considerations, for the sake of appearances.

## 7 A MORE SYSTEMATIC APPROACH TO INEQUITIES: THE CLIFF ANALOGY

Centering on inequities can create space for a systematic approach. Interventions that address inequities can be organized by employing a cliff analogy.^5^ People can be prevented from falling off a cliff by a fence (primary prevention), a net can catch people before they hit bottom (secondary prevention or condition treatment), or those who tumble all the way down can be carted away in an ambulance (tertiary prevention or amelioration of disease sequelae).^5^ Even better, people being pushed closest to the edge can be allowed to move back (addressing the social determinants of health) or at least protected by strengthening the fence in their area (prioritized primary prevention).^5^

The cliff analogy can help to understand the rationale for focusing on upstream causes of bad outcomes, and the need to focus resources on preventive care.^29^ Information about inequities (who is closest to the edge) is important for prioritizing access to primary prevention. Recommendations against fences (primary prevention, such as depression screening) might make sense for those furthest away from the edge or with alternative protections, but others are left in a precarious position.

Perhaps most importantly, the analogy shows the importance of involving people pushed close to the cliff's edge (those experiencing disadvantages or inequities) in drafting guidance. Although they play an important role, ambulance drivers or dispatchers (experts in diseases or guideline methods) cannot easily see how to stop people from falling – those pushed to the edge have a clear view.

## 8 MEASURING THE EFFECTS ON INEQUITIES

Of course, recommendations intended to promote health equity may not actually do so, just as clinical practice guidelines may not substantially improve or even change care.^30^ Structural issues and discriminatory practices^16^ could allow inequities to persist despite well formulated guidance. Powerful institutions, not just guidelines, will need to change to promote equity. The actual effects of guidance aimed at promoting health equity should be tracked and studied while recognizing that the effects of guidelines can be difficult to isolate from concomitant changes.

Health outcomes depend on income, gender, sexuality, racialization and other social factors.^31^ Although inequities in health outcomes are related to systemic unfairness that has a long history, health care can help to make health outcomes more fair.^31^, ^32^ At the same time, health care provided in unfair societies can propagate and exacerbate inequities.^33^ Guidance about how to address inequities through health care should not be misconstrued as suggesting that health care provision is the ultimate source of, or answer, to inequities.

## 9 REDEPLOYING FUNDING AND RESOURCES

Guidelines on certain topics seem to sprout up in multiple places while guidance on other important topics is missing or sparse. A more coordinated approach could involve certain topics prioritized for guidance by international organizations such as the World Health Organization. This guidance could be developed in support of other international processes such as the World Health Organization's model list of essential medicines that helps to determine which medicines are actually available to billions of people.

Prestigious guideline producers, such as national bodies, hand down recommendations for the general population, perhaps in the hope that others might adapt the recommendations for “special populations”. It is unclear if this actually happens and whether funding and capacity for applying guidance to people experiencing disadvantages exist. Flipping this around, well‐resourced guideline producers could do the more complicated work of providing guidance on the care of people experiencing specific disadvantages. Equity promotion could become a central or core activity if funding allocations flowed from grand statements about the importance of equity.

## 10 CONCLUSION

Clinical practice guidelines can easily encapsulate rather than address inequities. Guideline producers should select topics where guidance can actually address inequities and then use information about disparities to make helpful recommendations. Funders and journal editors should insist that guidelines explain how panels were formed and why new guidance on the topic is needed as, in some cases, equity might be advanced simply by refocusing or even cancelling planned guidelines. Changes to clinical practice guidelines will not be enough to promote health equity, but guidelines can be part of the solution if they are thoughtfully produced and acted on by clinicians and by governments.

## AUTHOR CONTRIBUTIONS

Not applicable.

## CONFLICT OF INTEREST STATEMENT

Nav Persaud reports funding from the Canadian Institutes of Health Research and the Ontario SPOR Support Unit. These funding sources had no role in the design, conduct, or reporting of the research described in this submission.

## ETHICS STATEMENT

Not applicable.

## Data Availability

Data sharing not applicable to this article as no datasets were generated or analysed during the current study.
